# Long-Term Exposure to Air Pollution Associates the Risk of Benign Brain Tumor: A Nationwide, Population-Based, Cohort Study in Taiwan

**DOI:** 10.3390/toxics10040176

**Published:** 2022-04-02

**Authors:** Kuang-Hsi Chang, Chieh-Lin Jerry Teng, Yi-Chao Hsu, Stella Chin-Shaw Tsai, Han-Jie Lin, Tsai-Ling Hsieh, Chih-Hsin Muo, Chung Y. Hsu, Ruey-Hwang Chou

**Affiliations:** 1 Department of Medical Research, Tungs’ Taichung MetroHarbor Hospital, Taichung 435, Taiwan; kuanghsichang@gmail.com (K.-H.C.); a92015t@gmail.com (T.-L.H.); 2 Center for General Education, China Medical University, Taichung 404, Taiwan; 3 General Education Center, Jen-Teh Junior College of Medicine, Nursing and Management, Miaoli 356, Taiwan; 4 Department of Life Sciences and Ph.D. Program in Translational Medicine, National Chung Hsing University, Taichung 402, Taiwan; 5 Division of Hematology/Medical Oncology, Department of Medicine, Taichung Veterans General Hospital, Taichung 407, Taiwan; drteng@vghtc.gov.tw; 6 Department of Life Science, Tunghai University, Taichung 407, Taiwan; 7 School of Medicine, Chung Shan Medical University, Taichung 402, Taiwan; 8 College of Medicine, National Chung Hsing University, Taichung 402, Taiwan; 9 Institute of Biomedical Sciences, Mackey Medical College, New Taipei City 252, Taiwan; hsuyc@mmc.edu.tw; 10 Department of Otolaryngology, Tungs’ Taichung MetroHarbor Hospital, Taichung 435, Taiwan; tsaistella111@gmail.com (S.C.-S.T.); commandity@gmail.com (H.-J.L.); 11 Rong Hsing Research Center for Translational Medicine, College of Life Sciences, National Chung Hsing University, Taichung 402, Taiwan; 12 Management Office for Health Data, China Medical University Hospital, Taichung 404, Taiwan; b8507006@gmail.com; 13 Graduate Institute of Clinical Medical Science, China Medical University, Taichung 404, Taiwan; hsucy63141@gmail.com; 14 The Ph.D. Program of Biotechnology and Biomedical Industry, China Medical University, Taichung 404, Taiwan; 15 Graduate Institute of Biomedical Sciences, China Medical University, Taichung 404, Taiwan; 16 Center for Molecular Medicine, China Medical University Hospital, Taichung 404, Taiwan; 17 Department of Medical Laboratory and Biotechnology, Asia University, Taichung 413, Taiwan

**Keywords:** air pollution, carbon monoxide (CO), nitrogen dioxide (NO_2_), particulate matter (PM), benign brain tumor (BBT)

## Abstract

Air pollutants as risk factors for benign brain tumor (BBT) remain unclear. Therefore, we conducted a nationwide retrospective cohort study by integrating the patients’ clinical data and daily air quality data to assess the environmental risk factors of BBT in Taiwan.Daily air quality data were categorized into quartiles (Q1 to Q4). The adjusted hazard ratio (aHR) was evaluated by comparing the BBT incidence rate of the subjects in Q2–Q4 with that of the subjects in Q1 (the lowest concentration of air pollutants). A total of 161,213 subjects were enrolled in the study. Among the air pollutants tested, the aHR of BBT was significantly higher in the subjects who were exposed to the highest level (Q4) of CO (aHR 1.37, 95% CI 1.08–1.74), NO_2_ (aHR 1.40, 95% CI 1.09–1.78), and PM_2.5_ (aHR 1.30, 95% CI 1.02–1.65) than that in the subjects who were exposed to the lowest level (Q1). No significant risk association of BBT with SO_2_ and PM_10_ exposure was observed. The results revealed that long-term exposure to air pollutants, particularly CO, NO_2_, and PM_2.5_, is associated with the risk of BBT.

## 1. Introduction

Ambient air pollution (also known as outdoor air pollution) has become a major global environmental issue that influences not only climate change but also human health [1]. Among all environmental and occupational risks, air pollution is the leading level 2 risk factor for disability-adjusted life years (DALYs). It was responsible for 213 million DALYs (95% uncertainty interval [UI]: 189–240) and 6.67 million deaths (UI 5·90–7·49) in 2019, according to the recent Global Burden of Diseases (GBD) report [2]. The burden of air pollution is related to human diseases and dysfunction, including respiratory diseases [3], cardiovascular diseases [4], reproductive [5], central nervous system dysfunctions [6], sensorineural hearing loss [7,8], psychiatric disorders [9,10], and cancers such as those of the lung, bladder, breast, nasopharyngeal [11,12], and brain tumors [13,14].

Brain tumors are mainly divided into primary and secondary brain tumors, according to their original lesion site. The former originates from the brain or its immediate surroundings and rarely spreads to distant organs, and the latter develops in another part of the body and metastasizes to the brain. Primary brain tumors are classified as benign (or non-malignant) and malignant brain tumors, the most common of which are meningioma (37.6%) and glioblastoma (14.6%), respectively. The 5-year survival rate was 91.5% in patients with benign brain tumors (BBTs) and 35.8% in those with malignant brain tumors [15]. Although the survival rate is relatively high, BBTs are life threatening because non-malignant cancer cells can damage and destroy normal brain tissue by continuously growing and spreading nearby, leading to an influence on normal brain functions. Several studies have explored numerous potential genetic risk factors for primary brain tumors, such as ATM serine/threonine kinase (ATM), cyclin dependent kinase inhibitor 2A (CDKN2A), isocitrate dehydrogenase (NADP(+)) 1/2 (IDH1/IDH2), erb-b2 receptor tyrosine kinase 2 (ERBB2, also named as HER2), neurofibromin 1 (NF1), NF2, moesin-ezrin-radixin-like (MERLIN) tumor suppressor (NF2), phosphatase and tensin homolog (PTEN), RB transcriptional corepressor 1 (RB1), tumor protein p53 (TP53), and germline genetic variants (SNPs, single nucleotide polymorphisms) [16,17]. Moreover, environmental risk factors are also associated with primary brain tumors. Among them, the increased risk of ionizing radiation (IR) and decreased risk with a history of allergies have been validated. Other environmental risk factors, including toxic agents (N-nitroso compounds, and pesticides), air pollution, and radiofrequency electromagnetic waves, have also been reported to be associated with primary brain tumors [14]. However, contradictory results were observed in ambient air pollution and the risk of brain tumors. Therefore, to clarify the association of air pollutants with the risk of BBT, we conducted a nationwide retrospective cohort study by integrating the patients’ clinical data from the Longitudinal Generation Tracking Database (National Health Insurance Database) and daily air quality data from the Taiwan Air Quality Monitoring Database (TAQMD) to assess the environmental risk factors of BBT in Taiwan.

## 2. Materials and Methods

### 2.1. Data Source

The longitudinal registry for beneficiaries, medical records of outpatients, inpatient visits, and catastrophic diseases of one million people were randomly selected from all insured beneficiaries in the National Health Insurance Research Database (NHIRD) from 1996 to 2011. Up to 99.99% of residents in Taiwan were enrolled in the National Health Insurance (NHI) program. Details of the NHIRD have been described elsewhere [18]. All datasets can be linked using unique encrypted personal identifiers. Disease diagnoses were defined by the International Classification of Diseases, Ninth Revision, Clinical Modification (ICD-9-CM).

Daily air quality data, including concentrations of sulfur dioxide (SO_2_; ppb), carbon monoxide (CO; ppm), nitrogen dioxide (NO_2_; ppb), particulate matter (PM): PM_10_ (μg/m^3^) and PM_2.5_ (μg/m^3^), were collected from the Taiwan Air Quality-Monitoring Database (TAQMD) on the Taiwan Environmental Protection Administration (EPA) website (https://airtw.epa.gov.tw/CHT/Query/His_Data.aspx; accessed on 31 May 2021) [19]. There were 83 EPA air monitoring stations in Taiwan. PM_2.5_ (μg/m^3^) data were monitored from 2005. Subsequently, the air quality data were linked to the corresponding residential townships of each subject.

### 2.2. Study Subjects

The subjects who lived in the area with air quality monitoring stations were identified. The living area for the individual insurant was defined based on the clinical visits for acute upper respiratory tract infection (AURTI; ICD-9 cm code 460). The study index date was 1 January 2000. The inclusion criteria were (1) those aged equal to or more than 20 years, (2) those ever diagnosed with acute upper respiratory tract infection (ICD-9 cm 460) during the study period, and (3) those with complete data on air pollutants and demographic information. The exclusion criteria were those with cancers (ICD-9 cm codes 140–208) before the study index date. Finally, 161,213 subjects were included in the study. All subjects were followed from the index date to the occurrence of BBTs (BBT; ICD-9 cm codes 225, 227.3, and 227.4), termination of insurance, or 31 December 2011, whichever came first.

### 2.3. Categorization of Urbanization and Insurance Levels

The level of urbanization was determined according to the population density (number of people/km^2^), population ratio of people with a college-level education or higher, population ratio of people aged over 65 years, population ratio of agricultural workers, and number of physicians per 100,000 people [20]. Urbanization is associated with medical convenience and air pollution. Insurance level was based on the personal income, and highly correlated with socio-economic status [21]. Thus, we considered the impact of urbanization and insurance levels to avoid the potential bias and overestimation of the risk of BBT.

### 2.4. Exposure Measurement

Each resident was assigned pollutant-exposure levels in accordance with the data obtained from the monitoring station located within his residential area. Due to the long latency period of BBT development, the daily average concentrations of air pollutants in each subject were calculated from 1998 to the end of the study. Air pollutant concentrations were categorized into quartiles from Q1 (lowest concentration) to Q4 (highest concentration).

### 2.5. Comorbidities

Comorbidities including diabetes mellitus (DM, ICD-9 cm code 250), ischemic heart disease (IHD, ICD-9 cm codes 410–414), hypertension (HT, ICD-9 cm codes 401–405), chronic obstructive pulmonary disease (COPD, ICD-9 cm codes 490–496), alcoholism (ICD-9 cm codes 303, 305.0, and V113), hyperlipidemia (ICD-9 cm code 272), and asthma (ICD-9 cm code 493) before the index date were considered as potential risk factors for BBT.

### 2.6. Statistical Analysis

We tested the age and follow-up year between BBT group and non-BBT group by performing Student’s *t*-test and tested the distribution of air pollutants by Man–Whitney test. The chi-square test was applied to examine the distribution of air pollutants among urbanization zones. A Cox regression model was used to calculate the hazard ratio (HR) and 95% confidence interval (CI). All models were adjusted for age, sex, insurance fee, urbanization, COPD, HT, IHD, asthma, DM, alcoholism, and hyperlipidemia. All statistical analyses were performed using SAS software (version 9.4; SAS Institute Inc., Cary, NC, USA).

## 3. Results

A total of 161,213 subjects were enrolled in the study. The baseline characteristics of all subjects and the average exposure concentrations of air pollutants during the follow-up period were analyzed. The results showed that the mean age of all subjects, including 44% of males and 56% of females, was 40.4 ± 14.6 years old. The mean follow-up periods of time were 8.0 ± 2.6 years and 11.7 ± 0.9 years in the BBT group and in the non-BBT group, respectively. The subjects with BBT were more in female (66.1%) than that in male (33.9%). More than half (67%) of the subjects lived in highly urbanized areas (the highest and the second highest urbanization). The residents with BBT had significant higher percentage of comorbidity including hypertension (HT) (22.2%), chronic obstructive pulmonary disease (COPD) (27.5%), hyperlipidemia (HL) (13.7%), and ischemic heart disease (IHD) (10.5%) than those with non-BBT group. The median concentrations of air pollutants were 4.32 ppb SO_2_, 0.68 ppm CO, 23.7 ppm NO_2_, 55.9 μg/m^3^ PM_10_, and 33.3 μg/m^3^ PM_2.5_, in which the concentration of CO was significant higher in the residents with BBT (Table 1).

The incidence rate (IR) and incidence rate ratio (IRR) of BBT in the residents exposed to each air pollutant categorized by quartile (Q1 to Q4 from the lowest to the highest) were determined. As shown in Table 2, the IRs of BBT in the residents exposed to Q1, Q2, Q3 and Q4 of each air pollutant were 0.30, 0.32, 0.25 and 0.26 for SO_2_, 0.28, 0.23, 0.26, and 0.36 for CO, 0.31, 0.29, 0.17 and 0.43 for NO_2_, 0.29, 0.23, 0.32 and 0.29 for PM_10_, and 0.25, 0.26, 0.31, and 0.30 for PM_2.5_, per 1000 person years, respectively. Compared to the residents exposed to the lowest concentration (Q1) of air pollutants, the IRRs of BBT were higher in those who were exposed to the highest concentration (Q4) of CO, NO_2_, and PM_2.5_ by 1.28, 1.37, and 1.23-folds, respectively.

The adjusted hazard ratios (aHRs) of BBT in the participants were further evaluated by adjusting age, gender, insurance levels, urbanization, and comorbidity including COPD, HT, IHD, asthma, diabetes, hyperlipidemia, and alcoholism. As shown in Table 3, among the air pollutants tested, the aHRs of BBT were significantly higher in the subjects who were exposed to the highest level (Q4) of CO (aHR 1.37, 95% CI 1.08–1.74), NO_2_ (aHR 1.40, 95% CI 1.09–1.79), and PM_2.5_ (aHR 1.29, 95% CI 1.02–1.65) than that in the subjects who were exposed to the lowest level (Q1). The results revealed that long-term exposure to air pollutants, particularly CO, NO_2_, and PM_2.5_, is associated with the risk of BBT.

## 4. Discussion

Traffic has been reported as the main contributor to urban air pollution. The major pollutants emitted from vehicles include CO and NO_2_, which adversely affect the environment [22,23]. Most heavy industries, such as the petrochemical, iron and steel, and coal-fired power plants, are in the rural areas of Taiwan. These industries have severe impacts on the air quality [24]. PM_2.5_ pollution is higher in a petrochemical industry city such as Zibo in China [25]. Approximately 70% of the primary PM_2.5_ was emitted from coal combustion, iron ore and steel industry, and non-ferrous metallurgy in central Taiwan [26]. Taken together, air pollution is associated with urbanization and industrialization [27,28].

In this study, we performed the multivariate analysis with two different variable types of each pollutant: continuous and category. The interquartile range is very small, which makes the differences of event number in each group not so significant. By the results from Table 3, the associations between the risk of BBT development and pollutants exposure were a potential non-linear correlation. There may be a specific threshold. However, this needs to be confirmed by further research.

The impact of air pollution on human health includes numerous diseases, such as those of respiratory, cardiovascular, and cancers [1]. Ambient air pollutants penetrate the respiratory system via inhalation, leading to the induction of low-grade and long-term inflammation and oxidative stress [29], and is associated with the risk of lung cancer, as well as other types of cancers [11]. Our study demonstrated that long-term exposure to air pollution was associated with the incidence of BBT. In particular, CO, NO_2_, and PM_2.5_ exposure associated with the risk of BBT (Table 2 and Table 3). Carbon monoxide (CO) is recognized as an exogenous toxic gas because of its higher binding affinity to hemoglobin than oxygen, which causes tissue hypoxia, oxidative stress, and multiple detrimental effects on cellular metabolism, oxygen utilization, the cardiovascular system, and neurocognitive processes [30,31]. Increased circulating carboxyhemoglobin (COHb) levels have been observed in patients with colorectal cancer through upregulation of heme oxygenase-1 (HO-1) [32]. The experimental results from the mouse model demonstrated that elevated COHb levels reduced the blood’s capacity to carry O_2_, leading to the induction of tumor hypoxia [33]. Hypoxia microenvironment contributes to tumor development, progression, and malignancy [34,35]. A recent report revealed that ambient CO exposure is associated with the risk of mortality in glioblastoma patients [36]. Nitrogen dioxide (NO_2_), a marker of traffic-sourced air pollution, has been reported to be associated with lung cancer mortality [37,38]. An animal study demonstrated that inhalation of ambient concentrations of NO_2_ facilitates blood-borne cancer cell spreading and colonization of the lungs by disturbing natural immunity in part [39]. Exposure to ambient NO_2_ levels reduced the CD4^+^ T-lymphocyte subpopulation in AKR/cum mice [40]. These results provide evidence that in vivo exposure to the concentrations commonly measured in the urbanization areas of NO_2_ negatively affects the immune system and is conducive to cancer progression. Sulfur dioxide has been considered as a risk factor of lung cancer [41,42], liver cancer [43,44], and gynecological cancer [45]. However, the association of SO_2_ with glioblastoma multiforme (GBM) was inconsistent. In a cohort from Severance Hospital in Korea, SO_2_ was associated with an elevated risk of GBM (HR 1.09; 95% CI: 1.026–1.159), whereas in the Surveillance, Epidemiology, and End Results Program (SEER) cohort from United States, no risk association of SO2 with GBM was observed (HR: 0.99; 95% CI: 0.983–0.998) [36]. Our current results revealed that SO_2_ was not associated with the incidence rate (Table 2) and risk (Table 3) of BBT. Fine particulate matter (PM_2.5_) contains numerous toxic compounds (e.g., acids and heavy metals). Owing to the small particle size, large numbers, and large surface area-to-mass ratio, PM_2.5_ can penetrate deeper into the lung and cause a more severe impact on health than the larger PM and windblown soil particle mass [46]. Long-term exposure to PM_2.5_ is associated with high mortality from respiratory disease, lung cancer, and cardiovascular disease [47], and reduction in PM_2.5_ levels is beneficial to lower cancer incidence [48]. Stimulation of PM_2.5_ on non-small cell lung cancer (NSCLC) cells, increased cell proliferation, migration, and invasion via upregulation of interleukin-1β (IL-1β) and matrix metalloprotease 1 (MMP1) [49]. Addition of exosomes from PM_2.5_-treated lung cancer cells promoted the growth of lung cancer cells through activation of the Wnt3a/β-catenin pathway [50]. Exposure to PM_2.5_ resulted in an increased number of tumor nodules and elevated expression of MMP1, IL-1β, VEGF, and angiogenesis factors in a tumor-bearing mouse model [51]. Ambient PM_2.5_-induced reactive oxygen species (ROS) were also involved in the progression of lung cancer [52]. A recent study demonstrated that chronic exposure to PM_2.5_ induces tumorigenesis and distant metastasis of lung adenocarcinoma by promoting epithelial-mesenchymal transition (EMT) and stem cell properties in patient-derived xenograft (PDX) models [53]. Long-term exposure to PM_2.5_, in addition to lung cancer, has been reported to be associated with the incidence of other cancer types, such as colorectal cancer [54,55], hepatocellular carcinoma [56], and malignant brain tumors [13,57]. Furthermore, the current study demonstrated that exposure to higher PM_2.5_ associated with the higher incidence rate of BBT (Table 2). The results from a 3D microfluidic platform of PM_2.5_-polluted human brain models revealed that PM_2.5_ can penetrate the blood–brain barrier (BBB) and accumulate in the brain tissue side, leading to astrogliosis, slight neuronal loss, and microglial infiltration. Exposure to PM_2.5_ induced the secretion of IL-1β and interferon-γ (IFN-γ) from neurons and reactive astrocytes, thereby promoting the infiltration of microglia into the M1 phenotype. M1 microglia release proinflammatory mediators and nitric oxide, which deteriorate neuronal damage [58]. It has been demonstrated that chronic exposure to ambient PM_2.5_ resulted in neuronal inflammation, leading to cognitive decline in the C57BL/6 mice model [59]. These studies provided the evidence and possible mechanisms of air pollution-induced neurodegenerative diseases in vivo. The smaller size of PM, the higher incidence rate of female lung cancer has been revealed. The incidence rate was greater for PM_1_ (5.98%), followed by PM_2.5_ (3.75%) and PM_10_ (1.57%) in female lung cancer [60]. Long-term exposure to PM_10_ was also reported to be associated with the risk of lung cancer [60,61] and other types of cancers such as mouth and throat cancer, non-melanoma skin cancer (NMSC), prostate cancer, and breast cancer [62]. Among the traffic-related air pollutants, PM_2.5_, but not PM_10_ was the dominant risk factor of malignant brain tumors [57]. Our results indicated that long-term exposure to PM_2.5_ associated with incidence rate (Table 2) and risk (Table 3) of BBT. Nevertheless, the underlying mechanisms of air pollution-associated carcinogenesis in brain tumors remain unclear and need further elucidation.

This study has several advantages. First, a relatively large sample size was investigated, followed by a long follow-up period (11.7 ± 0.9 years). Second, almost 99.99% of the residents in Taiwan were enrolled in the NHIRD [18], which minimized the bias of data collection, region, age, and institutions. Third, urbanization levels were included to evaluate the geographic association between the severity of air pollution and BBT. However, there are still some limitations to the study. The levels of air pollution in the residential areas of NHIRD insurants were determined by the closest air quality monitoring stations to clinics or hospitals. Consequently, the results may be biased, as measured air quality and urbanization levels may vary from actual values, especially when the participants commute long distances between their residential and equipment. Second, we did not have information on exposure to air pollution during work and commuting. Owing to the long latency of brain tumor, the exact exposure window might not affect the outcome of long-term events. Therefore, historical air pollution exposure is likely to be relevant to the etiology of BBT. Third, the genetic information of the study subjects was unavailable in the NHIRD. Some genetic risk factors, such as ATM, CDKN2A, IDH1/IDH2, NF1, NF2, PTEN, RB1, and TP53, have been reported to be associated with brain tumors [16]. These genetic variables were not adjusted or were excluded from the study. Fourth, detailed information on other potential confounding factors, such as family history, systemic comorbidities, smoking or alcohol drinking, dietary factors, and socioeconomic status, were not recorded in the NHIRD. Some potential risk factors for BBT may be missing. Despite these limitations, the impacts of biases might be reduced or negligible in the current nationwide study with a long follow-up period.

## 5. Conclusions

In conclusion, we integrated the patients’ clinical data and daily air quality data to assess the environmental risk factors of BBT. The residential area of the patients was redefined by the location of the hospital or clinics. The aHR of BBT was used to evaluate the risk of air pollutants. The aHR of BBT was significantly higher in the subjects exposed to the highest levels of CO, NO_2_, and PM_2.5_ compared to subjects exposed to the lowest level. The results indicated that long-term exposure to air pollutants, particularly CO, NO_2_, and PM_2.5_, is associated with the risk of BBT. However, further detailed experiments and clinical studies are needed to validate these findings.

## Figures and Tables

**Table 1:** Distribution of the demographic data, comorbidities, and air pollutants of the study participants.

Variables	BBT (*n* = 531)	Non- BBT (*n* = 160,682)	*p*-Value	Total (*n* = 161,213)
Age, years				
Mean (SD)	45.0 (14.5)	40.4 (14.6)	<0.001	40.4 (14.6)
Follow-up years				
Mean (SD)	8.0 (2.6)	11.7 (0.9)	<0.001	11.7 (0.9)
Sex *n* (%)				
Male	180 (33.9)	70,733 (44.0)	<0.001	70,913 (44.0)
Female	351 (66.1)	89,949 (56.0)		90,300 (56.0)
Insurance levels, *n* (%)				
Low	72 (13.6)	24,777 (15.4)	0.444	24,849 (15.4)
2nd	274 (51.6)	82,570 (51.4)		82,844 (51.4)
High	185 (34.8)	53,335 (33.2)		53,520 (33.2)
Urbanization, *n* (%)				
Highest	194 (36.5)	55,367 (34.5)	0.483	55,561 (34.5)
2nd	156 (29.4)	52,235 (32.5)		52,391 (32.5)
3rd	92 (17.3)	27,252 (17.0)		27,344 (17.0)
Lowest	89 (16.8)	25,828 (16.1)		25,917 (16.1)
Comorbidity, *n* (%)				
Hypertension (HT)	118 (22.2)	21,648 (13.5)	<0.001	21,766 (13.5)
Chronic Obstructive Pulmonary Disease (COPD)	146 (27.5)	29,449 (18.3)	<0.001	29,595 (18.4)
Hyperlipidemia (HL)	73 (13.7)	13,092 (8.1)	<0.001	13,165 (8.2)
Diabetes	31 (5.8)	6825 (4.2)	0.088	6856 (4.3)
Alcoholism	6 (1.1)	1768 (1.1)	1.000	1774 (1.1)
Ischemic heart disease (IHD)	56 (10.5)	9732 (6.1)	<0.001	9788 (6.1)
Asthma	15 (2.8)	2921 (1.8)	0.116	2936 (1.8)
Median concentration of air pollutants, median (interquartile range)				
SO_2_ (ppb)	4.16 (3.32, 5.59)	4.32 (3.38, 6.03)	0.031 ^#^	4.32 (3.38, 6.03)
CO (ppm)	0.70 (0.54, 0.87)	0.68 (0.56, 0.81)	0.013 ^#^	0.68 (0.56, 0.81)
NO_2_ (ppm)	23.1 (18.2, 28.0)	23.7 (18.2,27.5)	0.152 ^#^	23.7 (18.2, 27.5)
PM_10_ (μg/m^3^)	61.6 (51.5, 69.2)	55.9 (51.6, 68.6)	0.427 ^#^	55.9 (51.6, 68.6)
PM_2.5_ (μg/m^3^)	34.8 (29.5, 41.6)	33.3 (29.5, 41.2)	0.094 ^#^	33.3 (29.5, 41.2)

BBT: benign brain tumor. #: *p*-value for Mann–Whitney test.

**Table 2:** The incidence rates (IRs) and crude incidence rate ratios (IRRs) of BBT in the residents exposed to each quartile of air pollutants.

Levels		*n* of BBT	PY	IR	IRR	95%CI	
SO_2_ (ppb)							
Q1	<3.38	147	490,768	0.30	1 (reference)		
Q2	3.38–4.31	142	446,068	0.32	1.06	0.84	1.34
Q3	4.32–6.0	135	541,763	0.25	0.83	0.66	1.05
Q4	>6.0	107	407,458	0.26	0.88	0.68	1.12
CO (ppm)							
Q1	<0.56	131	468,600	0.28	1 (reference)		
Q2	0.56–0.68	111	489,449	0.23	0.81	0.63	1.04
Q3	0.69–0.81	109	423,067	0.26	0.92	0.72	1.19
Q4	>0.81	180	504,940	0.36	1.28	1.02	1.60
NO_2_ (ppm)							
Q1	<18.21	131	419,033	0.31	1 (reference)		
Q2	18.21–23.68	143	492,285	0.29	0.93	0.73	1.18
Q3	23.69–27.48	107	622,574	0.17	0.55	0.42	0.71
Q4	>27.48	150	352,164	0.43	1.37	1.08	1.73
PM_10_ (μg/m^3^)							
Q1	<51.58	136	465,897	0.29	1 (reference)		
Q2	51.58–55.92	109	469,090	0.23	0.79	0.62	1.02
Q3	55.93–68.57	151	478,478	0.32	1.08	0.86	1.36
Q4	>68.57	135	472,593	0.29	0.98	0.77	1.24
PM_2.5_ (μg/m^3^)							
Q1	<29.46	135	541,674	0.25	1 (reference)		
Q2	29.46–33.30	110	416,298	0.26	1.06	0.82	1.36
Q3	33.31–41.22	142	455,475	0.31	1.25	0.99	1.58
Q4	>41.22	144	472,611	0.30	1.23	0.97	1.55

*n* of BBT: number of patients with benign brain tumor. PY: person years. IR: incidence rate (per 1000 person years). IRR: incidence rate ratio.

**Table 3:** Adjusted hazard ratios (aHRs) of BBT in the residents exposed to the highest, 2nd, and 3rd concentrations of air pollutants compared to those exposed to the lowest concentration.

Pollutants	Levels	Adj. HR	95%CI		*p*-Value
SO_2_ (ppb)	Continuous	0.974	0.938	1.012	0.184
Q1	<3.38	1 (reference)			
Q2	3.38–4.31	1.06	0.84	1.33	0.637
Q3	4.32–6.0	0.85	0.67	1.08	0.177
Q4	>6.0	0.91	0.71	1.18	0.495
CO (ppm)	Continuous	1.73	1.27	2.34	<0.001
Q1	<0.56	1 (reference)			
Q2	0.56–0.68	0.87	0.68	1.13	0.299
Q3	0.69–0.81	0.99	0.77	1.29	0.957
Q4	>0.81	1.37	1.08	1.74	0.009
NO_2_ (ppm)	Continuous	1.013	0.999	1.027	0.077
Q1	<18.21	1 (reference)			
Q2	18.21–23.68	0.97	0.76	1.23	0.787
Q3	23.69–27.48	0.57	0.44	0.74	<0.001
Q4	>27.48	1.40	1.09	1.79	0.007
PM_10_ (μg/m^3^)	Continuous	1.003	0.996	1.009	0.409
Q1	<51.58	1 (reference)			
Q2	51.58–55.92	0.85	0.66	1.09	0.195
Q3	55.93–68.57	1.16	0.92	1.47	0.211
Q4	>68.57	1.04	0.82	1.33	0.741
PM_2.5_ (μg/m^3^)	Continuous	1.01	1.001	1.021	0.034
Q1	<29.46	1 (reference)			
Q2	29.46–33.30	1.10	0.85	1.41	0.474
Q3	33.31–41.22	1.32	1.04	1.68	0.023
Q4	>41.22	1.29	1.02	1.65	0.036

Adj. HR: adjusted hazard ratio in the multivariate analysis after adjusting for age, gender, insurance fee, urbanization, COPD, HT, IHD, asthma, DM, alcoholism and hyperlipidemia.

## Data Availability

Data are available from the NHIRD published by Taiwan National Health Insurance Bureau. Due to the ‘Personal Information Protection Act’, data cannot be made publicly available (http://nhird.nhri.org.tw/en/index.html; accessed on 1 December 2021).
